# Generation and Characterization of Rat and Mouse Monoclonal Antibodies Specific for MeCP2 and Their Use in X-Inactivation Studies

**DOI:** 10.1371/journal.pone.0026499

**Published:** 2011-11-28

**Authors:** K. Laurence Jost, Andrea Rottach, Manuela Milden, Bianca Bertulat, Annette Becker, Patricia Wolf, Juan Sandoval, Paolo Petazzi, Dori Huertas, Manel Esteller, Elisabeth Kremmer, Heinrich Leonhardt, M. Cristina Cardoso

**Affiliations:** 1 Department of Biology, Technische Universität Darmstadt, Darmstadt, Germany; 2 Department of Biology II, Ludwig Maximilians University Munich, Planegg-Martinsried, Germany; 3 Cancer Epigenetics and Biology Program, Bellvitge Biomedical Research Institute (IDIBELL), Barcelona, Spain; 4 Helmholtz Center Munich, German Research Center for Environmental Health, Institute of Molecular Immunology, Munich, Germany; Deutsches Krebsforschungszentrum, Germany

## Abstract

Methyl CpG binding protein 2 (MeCP2) binds DNA, and has a preference for methylated CpGs and, hence, in cells, it accumulates in heterochromatin. Even though it is expressed ubiquitously MeCP2 is particularly important during neuronal maturation. This is underscored by the fact that in Rett syndrome, a neurological disease, 80% of patients carry a mutation in the *MECP2* gene. Since the *MECP2* gene lies on the X chromosome and is subjected to X chromosome inactivation, affected patients are usually chimeric for wild type and mutant MeCP2. Here, we present the generation and characterization of the first rat monoclonal MeCP2 specific antibodies as well as mouse monoclonal antibodies and a rabbit polyclonal antibody. We demonstrate that our antibodies are suitable for immunoblotting, (chromatin) immunoprecipitation and immunofluorescence of endogenous and ectopically expressed MeCP2. Epitope mapping revealed that most of the MeCP2 monoclonal antibodies recognize the C-terminal domain and one the N-terminal domain of MeCP2. Using slot blot analysis, we determined a high sensitivity of all antibodies, detecting amounts as low as 1 ng of MeCP2 protein. Moreover, the antibodies recognize MeCP2 from different species, including human, mouse, rat and pig. Lastly, we have validated their use by analyzing and quantifying X chromosome inactivation skewing using brain tissue of MeCP2 heterozygous null female mice. The new MeCP2 specific monoclonal antibodies described here perform well in a large variety of immunological applications making them a very valuable set of tools for studies of MeCP2 pathophysiology *in situ* and *in vitro*.

## Introduction

**Figure 1:**
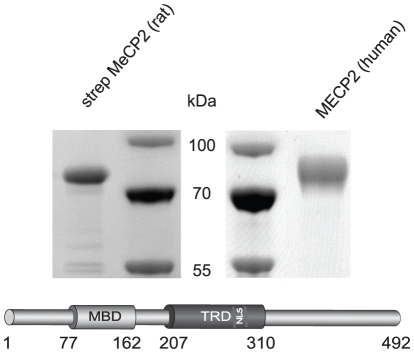
Antigen preparation. Purified strep-tagged MeCP2 (rat) and purified intein tagged MECP2 (human) were subjected to a SDS-PAGE and stained with Coomassie. The molecular weight markers are labeled in the middle. A schematic representation of the rat MeCP2 protein and its functional domains is shown below. MBD: methyl CpG binding domain; TRD: transcriptional repression domain; NLS: nuclear localization signal.

Methyl CpG binding protein 2 (MeCP2) was the second methyl CpG binding protein to be discovered [1] and the first to be cloned [2]. In interphase mouse nuclei, MeCP2 is prominently localized at heterochromatic foci [2]. In metaphase chromosomes, the association of MeCP2 with euchromatic arms is rather weak compared to a strong localization at pericentric heterochromatin [2], highly enriched in heavily methylated major satellite DNA repeats [3]. MeCP2 consists of a conserved methyl CpG binding domain (MBD) that binds to 5-methyl cytosine with high affinity and is shared with the other MBD protein family members. The transcriptional repression domain (TRD), which carries a nuclear localization sequence interacts with histone deacetylases and the transcriptional corepressor Sin3A [4], [5], [6]. Finally, the C-terminal domain binds nucleosomes (Figure 1).

Even though MeCP2 is ubiquitously expressed, it is genetically linked to a neurological disease called Rett syndrome (RTT, OMIM 312750). RTT was first described in 1966 by Andreas Rett [7] and affects one in every 10,000–15,000 female births [8], [9], [10]. Affected girls seem to develop normally until six to 18 months, subsequently they enter a developmental arrest, which is followed by strongly impaired motor skills, stereotypic hand movements, loss of speech, seizures, abnormal breathing, microcephaly, ataxia and other symptoms. Mutations within the *MECP2* gene located on chromosome Xq28 are found in approximately 80% of all classic RTT cases [8], [11]. Since *MECP2* is located on the X chromosome it is subjected to random X chromosome inactivation. Thus, depending on which chromosome was inactivated, a mosaic pattern of healthy (wild type allele expressing) and affected (mutant allele expressing) cells is created [12]. A further important aspect is the stark discrepancy between MeCP2 mRNA expression levels compared to protein levels (e.g. [13]), which highlights the need for highly specific antibodies detecting MeCP2 on a protein level.

Up to now rabbit polyclonal and mouse monoclonal antibodies have been raised against MeCP2 but the available antibodies are limited in their application range. Here, we describe the generation of the first rat monoclonal antibodies against MeCP2 being capable of reacting specifically in most common immunological applications. To complete the collection, we generated two mouse monoclonal antibodies and a rabbit polyclonal antibody. We could demonstrate the suitability of these high affinity and specific antibodies for immunoblotting, (chromatin) immunoprecipitation, and immunofluorescence stainings of cells and tissues. Additionally, we used one of our anti-MeCP2 rat monoclonal antibodies on MeCP2 heterozygous null mouse brain to analyze and quantify X chromosome inactivation skewing.

## Materials and Methods

### Plasmids

Mammalian expression constructs (Figure 3 and S1A) coding for GFP or YFP-tagged rat MeCP2 full length (MeCP2G) and domain constructs (MeCP2Y.3 and MeCP2Y.5) were previously described [14], [15]. The mammalian expression constructs MeCP2G.9 and MeCP2G.8 were generated from the above plasmids by PCR amplification using the following primers:

pMeCP2G.9 ss ccgctcgaggccatggggagcccttccaggagagaaca

 as cgcggatccttccgggtcttgcgcttcttgatggggagcac

pMeCP2G.8 ss ggaagatctgccatggaaaccgtcagcattgaggtcaag

 as ataagaatgcggccgcttacttgtacagctcgtccatgcc

**Figure 2:**
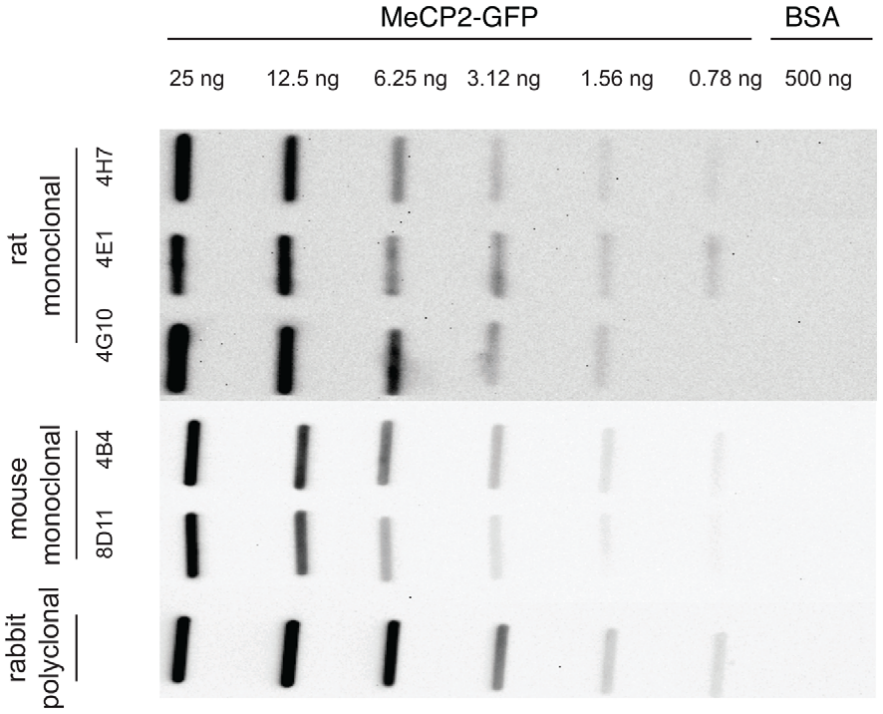
Antibody sensitivity. The detection limit of the MeCP2 antibodies was tested on native rat MeCP2-GFP by slot blotting analysis and lies between 1.58 and 0.78 ng of recombinant purified rat MeCP2.

The mammalian expression construct (Figure 6 and S1B) expressing GFP-tagged human MECP2 was described before [16] and was provided by S. Kudo (Hokkaido Institute of Public Health, Sapporo, Japan). For expression in Sf9 (Invitrogen Paisley PA4 9RF, UK) insect cells the Bac-to-Bac baculovirus expression system (Invitrogen Paisley PA4 9RF, UK) was used. To express MeCP2 with a N-terminal double strep-tag (Figure 1), a sequence encoding the strep-tactin target peptide strep tag III (MWSHPQFEKGGGSTGGGSGGGSWSHPQFEK) was synthesized (Entelechon, Bad Abbach, Germany) flanked by BamHI and NotI sites and subcloned into pFastBac1 (Invitrogen, Paisley PA4 9RF, UK) using the same sites. Rat MeCP2 full length was generated by PCR amplification from MeCP2G (described above) using the following primers:

MeCP2 ss: ggaagatctgccatggaaaccgtcagcattgaggtcaag

 as: ataagaatgcggccgcttacttgtacagctcgtccatgcc

with NotI and XhoI sites and subcloned in frame with the strep-tag in the pFastBac1 vector. For expression of rat MeCP2-GFP in Sf9 insect cells (Figure 2) the mammalian expression construct coding for MeCP2G full length (described above) was cut using NotI and XhoI and cloned in frame in the pFastBac1 vector.

A prokaryotic expression construct coding for intein tagged human MECP2 (Figure 1) [17] was obtained from C.L. Woodcock (University of Massachusetts, Amherst, USA).

### Tissues

Male mouse MeCP2 hemizygous brains (Figures 5 and 6) [18] were kindly provided by the group of P. Huppke (Georg August University, Göttingen, Germany). Female mouse MeCP2 null heterozygous brains (Figure 7) [18] were kindly provided by the group of L. Villard (Faculte de Medecine La Timone, Marseille, France). Wild type mouse brains (C57BL/6N; Charles River Laboratories International, Inc., Wilmington, MA 01887, USA) were used as control. Mice were over 10 months old.

### Cell culture and transfection

For immunofluorescence (IF) experiments mouse C2C12 myoblasts [19] were cultured using standard conditions described previously [20]. For subsequent IF experiments (Figure 6 and S1B), C2C12 cells were transiently transfected with human GFP-MECP2 expression construct [16] using Transfectin (Bio Rad, München, Germany) according to manufacturer's advice.

**Figure 3:**
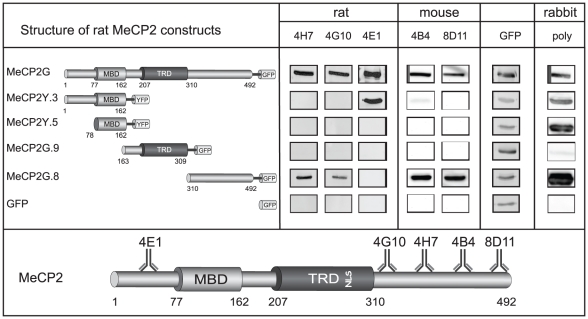
Epitope mapping. To determine the binding site of the new monoclonal antibodies within the MeCP2 protein, we probed extracts of mammalian cells expressing different MeCP2 constructs fused to GFP/YFP as indicated. To control for the level of the fusion proteins, the membranes were reprobed with anti GFP mouse monoclonal antibody. A summary of the epitope mapping results for the different antibodies is shown below. MeCP2 functional domains are as in Figure 1.

For the epitope mapping (Figure 3 and S1A), human embryonic kidney (HEK) 293T [21] cells were cultured in DMEM supplemented with 10% fetal calf serum and 50 µg/ml gentamicin and transfected with full length rat MeCP2 and domain constructs (described above) using polyethylenimine (Sigma, St. Louis, MO, USA).

MeCP2 antigens for immunization (Figure 1) and slot blot applications (Figure 2) were produced using the baculovirus system in Sf9 insect cells. Sf9 cells were maintained in EX-CELL 420 Insect Serum Free (SAFC) medium supplemented with 10% fetal bovine serum shaking at 100 rpm and 28°C. Transfection of Sf9 cells to produce recombinant baculovirus, was performed using Cellfectin (Invitrogen, Paisley PA4 9RF, UK) according to the manufacturer's instructions.

### Antigen purification

Sf9 insect cells were infected with the recombinant baculovirus (coding for MeCP2 with N-terminal double strep-tag; [22]) and incubated at 28°C with shaking for 5 days. The cells were pelleted by centrifugation (200× g, 5 min, 4°C) and resuspended in a buffer containing 25 mM Tris-HCl, pH 8.0; 1 M NaCl; 50 mM glucose; 10 mM EDTA; 0.2% Tween-20; 0.2% NP40. The buffer was supplemented with protease inhibitors (Complete mini; Roche, Mannheim, Germany). After incubation on ice for 10 min, cells were disrupted with a high-pressure homogenizer (EmulsiFlex-C5, Avestin) followed by centrifugation at 14,000× g for 30 min.

Strep-tagged recombinant rat MeCP2 protein was purified by incubating the supernatant with 500 µl of strep-tactin sepharose beads (IBA, Göttingen, Germany) for 4 h at 4°C on a rotary shaker. To elute strep-tagged proteins, the beads were incubated with D-Desthiobiotin (0.5 mg/ml; IBA, Göttingen, Germany), dissolved in 1× PBS, for 30 min at 4°C. After centrifugation (200× g, 2 min), beads were separated from the eluate containing the purified proteins. The elution step was performed three successive times.

Intein tagged human MECP2 protein was purified as previously described resulting in untagged MECP2 through protein splicing [23].

### Immunizations, generation of hybridomas and ELISA screening

Monoclonal antibodies specific for MeCP2 were generated via the hybridoma technology as described by Rottach et al. [24]. 80 µg of a N-terminal, strep-tagged full length rat MeCP2 were injected both intraperitoneally and subcutaneously into Lou/C rats and CBL mice using CpG2006 (TIB MOLBIOL, Berlin, Germany) as adjuvant. 8 weeks later and 3 days before fusion a boost was given intraperitoneally and subcutaneously. Spleen cells were isolated and fused to the myeloma cell line P3X63-Ag8.653 (ATCC, Rockville, MD, USA) using polyethylene glycol 1500 (PEG 1500, Roche, Mannheim, Germany). After fusion, cells were cultured in 96-well plates using RPMI 1640 with 20% fetal calf serum, penicillin/streptomycin, glutamine, pyruvate, and non-essential amino acids (PAA, Cölbe, Germany) supplemented by aminopterin (Sigma, St. Louis, MO, USA). The hybridoma supernatants were tested in a solid-phase enzyme linked immunosorbent assay (ELISA). Microtiter plates were coated over night with strep-tagged rat MeCP2 at a concentration of 3–5 g/ml in 0.1 M sodium carbonate buffer (pH 9.6) and blocked with non-fat milk (Frema, Neuform, Zarrentin, Germany). The hybridoma supernatants were added and the bound monoclonal antibodies were detected using a cocktail of biotinylated mouse monoclonal antibodies against the rat IgG heavy chains, thus avoiding the detection of IgM mouse monoclonal antibodies (anti IgG1, anti IgG2a, anti IgG2b [ATCC, Manassas, VA], anti IgG2c [Ascenion, Munich, Germany]). For visualization, peroxidase-labeled avidin (Alexis, San Diego, CA) antibodies were applied and o-phenylenediamine was used as chromogen in the peroxidase reaction. The clones 4H7, 4G10 and 4E1 (rat monoclonal) as well as 4B4 and 8D11 (mouse monoclonal) were stably subcloned and used for further characterization.

The rabbit polyclonal antibody was generated using the untagged human MECP2 according to the Express rabbit protocol from PickCell (PickCell, Amsterdam, Netherlands) and used in form of antiserum.

All monoclonal antibodies are available upon request.

### Ethics statement

Immunizations of mice and rats for the purpose of generating monoclonal antibodies were approved by the Government of Upper Bavaria, according to the animal experimentation law § 8a, permit number 209.1/211-2531.6-4/99.

### Sensitivity assay via slot blot analysis

#### Purification of MeCP2-GFP

Sf9 insect cells were infected with the recombinant baculovirus (coding for rat MeCP2-GFP) and incubated at 28°C with shaking for 5 days. The cells were pelleted and resuspended as explained above for strep-tag MeCP2 and disrupted by sonication (three times each for 25 seconds, 70% power; Bandelin Sonopuls GM70, Sonontrode HD70, Berlin, Germany) on ice. Lysates were cleared by centrifugation at 15,000× g for 30 min at 4°C.

Recombinant rat MeCP2-GFP protein was purified by incubating 200 ml whole cell lysate with 1 ml (1.5 mg/ml) GBP nanotrap according to the manufacturer's advice (Chromotek, Planegg-Martinsried, Germany). After transfer of the GBP nanotrap beads containing lysate to a Bio-Rad Poly-Prep chromatography column (Cat: 731-1550, Bio-Rad Laboratories, Hercules CA 94547, USA) the column was washed three times with 10 ml PBS. To elute the MeCP2-GFP protein, the beads were incubated with 5 ml of a high salt buffer. Buffer exchange was done with PBS using Amicon ultra centrifugal filters (Ultracel 10 kDa molecular weight cutoff; Millipore, Ireland). Eluted protein was quantified with Pierce the 660 nm protein assay (Thermo Scientific; Pro: #1861426, Schwerte, Germany) and checked by SDS-PAGE analysis (data not shown).

#### Slot blotting analysis

Native MeCP2-GFP was spotted directly onto a nitrocellulose membrane (GE Healthcare, München, Germany). Membranes were incubated in blocking buffer, 5% (w/v) non-fat dry milk in PBS (PBSM), for 20 min at room temperature. Primary antibodies were used undiluted and incubated for 2 h at room temperature, followed by three washes in PBS/0.1% Tween-20. Subsequently, membranes were incubated for 1 h at room temperature with horseradish peroxidase conjugated anti-rabbit IgG (Sigma, St. Louis, MO, USA) diluted 1∶10,000 or anti-mouse (GE Healthcare, München, Germany) and rat IgG (Sigma, St. Louis, MO, USA) 1∶5,000 in 5% (w/v) PBSM. After three washing steps in PBS/0.1% Tween-20, signals were detected with ECL (GE Healthcare, München, Germany).

### Epitope mapping

For epitope mapping, different constructs of rat MeCP2 with C-terminal GFP or YFP tag were used for transient transfection of HEK 293T cells. After cell lysis (20 mM Tris-HCl pH 7.5, 150 mM NaCl, 0.5%NP40, 2 mM PMSF, 0.5 mM EDTA, 1× mammalian protease inhibitor mix, 1 mg/ml DNase, 2 mM MgCl_2_) the concentration of the GFP fusion proteins was calculated using a fluorescent read out of GFP and YFP (Infinite® M1000, TECAN), respectively (GFP: excitation wavelength: 490 nm, emission wavelength: 511 nm, YFP: excitation wavelength: 525 nm, emission wavelength: 538 nm). The protein concentration was normalized to the construct with the lowest expression rate and lysates were diluted accordingly (228 nM GFP or YFP). The samples were boiled in Laemmli sample buffer at 95°C for 10 min and loaded on a 10% SDS-PAGE. Western blot analysis was performed as described above. In addition to the polyclonal and monoclonal anti MeCP2 antibodies, anti GFP mouse monoclonal antibody (Cat: 11814460001, Roche Diagnostics GmbH, Mannheim, Germany) was used to control for expression level of the different deletion proteins.

### Cross-species reactivity assay via western blot analysis

For western blot analysis brain cell nuclei were extracted from pig (obtained fresh from the local slaughterhouse), mouse and rat (Charles River Laboratories International, Inc., Wilmington, MA 01887, USA) as described [25] and lysed in RIPA buffer (50 mM Tris/HCl pH 8, 150 mM NaCl, 1% Tween, 0.5% Doc, 0.1% SDS). For each gel lane, lysates from 10^6^ nuclei were loaded.

Samples were separated on a 10% SDS-PAGE and transferred to a nitrocellulose membrane (GE Healthcare, München, Germany). The following primary antibodies were used for western blot analysis: rabbit polyclonal anti MeCP2 (1∶500), mouse monoclonal and rat monoclonal anti MeCP2 (undiluted). Secondary antibodies were as above for slot blot analysis.

### Immunoprecipitation

Mechanically disrupted mouse brain tissue (3–4 brains) was dissolved in buffer A (20 mM Tris pH 7.9, 0.6 M NaCl, 1.5 mM MgCl_2_, 0.2 mM EDTA, 0.4% NP-40), and then diluted with buffer B (20 mM Tris pH 7.9, 1.5 mM MgCl_2_, 0.2 mM EDTA, 0.4% NP-40) to obtain an NaCl concentration of 200 mM. Mouse brain extracts were incubated with 400 µl of the rat monoclonal MeCP2 antibody indicated, at 4°C for 2 h with shaking. As negative control, anti RFP mix rat monoclonal antibody [26] of equal amount was used. 100 µl protein G agarose beads, that were equilibrated with buffer B, were added and incubated with the extract for 1 h at 4°C with shaking. After three washes with buffer B immuno complexes were dissolved in 60 µl 1× Laemmli sample buffer.

For successful immunoprecipitation using the mouse monoclonal antibodies nuclei had to be isolated first, followed by a modified protocol. Mechanically disrupted mouse brain tissue was resuspended and washed with ice-cold PBS supplemented with protease inhibitor cocktail (Roche, Mannheim, Germany). Cell pellets were resuspended in cell lysis buffer (HEPES 5 mM, KCl 85 mM, NP40 0.5% pH 8.0) supplemented with protease inhibitor cocktail and subsequently homogenized with a douncer. The nuclear pellet obtained was resuspended in nuclei lysis buffer (Tris-HCl 50 mM, EDTA 10 mM, SDS 1% pH 8.1) and sonicated with a Bioruptor (Diagenode) for 5 minutes (30 sec ON, 30 sec OFF cycles) to get a homogeneous extract.

The extract was diluted with immunoprecipitation buffer (SDS 0.1%, Triton X-100 1.1%, EDTA 1.2 mM, NaCl 165 mM, Tris-HCl 16.7 mM pH 8.1) and followed by a pre-clearing overnight at 4°C with magnetic beads (Invitrogen, Paisley PA4 9RF, UK). Non-related mouse IgG antibody was used as a negative control. The amount of extract that was used for each immunoprecipitation varied from 10–500 µg. Antibody-Dynabeads M-280 sheep anti-mouse IgG (Invitrogen, Paisley PA4 9RF, UK) were added to pre-cleared chromatin and incubated with shaking for 2 h at 4°C. 10 µg of nuclear extract were loaded as input. After three washes with immunoprecipitation buffer immuno complexes were eluted from the beads with 25 µl 2× Laemmli sample buffer.

Samples were analyzed by western blot as described above.

### Chromatin immunoprecipitation

Nuclei were obtained from wild type and MeCP2 knock out male brain as described above with an additional cross-linking step, after mechanical disruption, with 1% formaldehyde for 10 min. Adding glycine to a final concentration of 0.125 M stopped the cross-linking reaction. The lysed nuclear pellet was sonicated with a Bioruptor (Diagenode) for 15 minutes (30 sec ON, 30 sec OFF cycles). The average chromatin size of the fragments obtained was ∼300 bp. Magnetic beads were used for pre-clearing of diluted chromatin (over night at 4°C) and for incubation (2 h at 4°C with shaking) with H3 (ab1791, Abcam), our rat monoclonal antibodies: 4E1, 4H7, and 4G10 or our mouse monoclonal antibodies: 4B4 and 8D11. Non-related mouse IgG antibody was used as a negative control. The input was obtained from the nuclear extract and represents 5% of the chromatin that is used for the chromatin immunoprecipitation with each antibody. The immuno-complexes were washed: twice with low salt buffer (Tris-HCl 50 mM pH 8.0, NaCl 150 mM, SDS 0.1%, NP-40 1%, EDTA 1 mM, deoxicolate Na 0.5%), twice with high salt buffer (Tris-HCl 50 mM pH 8.0, NaCl 500 mM, SDS 0.1%, NP-40 1%, EDTA 1 mM, Na deoxicolate 0.5%), twice with LiCl buffer (Tris-HCl 50 mM pH 8.0, LiCl 250 mM, SDS 0.1%, NP-40 1%, EDTA 1 mM, Na deoxicolate 0.5%) and twice with TE buffer (Tris-HCl 10 mM pH 8.0, EDTA 0.25 mM). Cross-linked chromatin was then eluted from the magnetic beads (Dynabeads M-280 sheep anti-mouse IgG for mouse monoclonal antibodies and protein G for rat monoclonal antibodies) by adding elution buffer (NaHCO_3_ 100 mM, SDS 1%). Samples were reverse cross-linked over night at 65°C and incubated with proteinase K at 50 µg/ml final concentration for 1 h. DNA was purified with the PCR purification kit (Qiagen) and used for PCR analysis, which was carried out with the following primers:

*Xist* (promoter) 5′-CCTGTACGACCTAAATGTCC-3′

 5′-GTATTAGTGTGCGGTGTTGC-3′.

In the case of our rat monoclonal antibodies 38 PCR cycles were used and in the case of our mouse monoclonal antibodies 35 cycles.

### Immunofluorescence analysis

#### Cells

For immunofluorescence staining, C2C12 cells were seeded on glass coverslips and transiently transfected with GFP tagged MECP2 (human). Cells were fixed with 3.7% formaldehyde in PBS and incubated with the undiluted rat/mouse anti MeCP2 antibodies for 1 h at room temperature. After incubation with the secondary Alexa 647 conjugated goat anti rat/mouse IgG antibody (Invitrogen Paisley PA4 9RF, UK) diluted 1∶400 in PBS containing 2% BSA, the cells were counterstained with DAPI (2 µg/ml) and mounted in Vectashield medium (Vector Labs, Burlingame, CA, USA).

#### Tissues

Mouse brains were fixed by overnight immersion in PBS-buffered 4% paraformaldehyde. The brains were embedded in Tissue Tek (Sakura, Zoeterwoude, Netherlands) and cryosectioned (25 µm) using a cryostat HM 560 (Microm, Walldorf, Germany).

Sections were air dried at room temperature for 30 min, re-hydrated in 10 mM sodium citrate buffer (pH 6.0) for 5 min, pulse-heated (80°C) for 30 min in the microwave. The slides were equilibrated in PBS after heating and incubated with the following antibodies: anti MeCP2 mouse monoclonal (undiluted), rat monoclonal (undiluted), rabbit polyclonal (1∶500), anti B23 mouse monoclonal (Sigma, St. Louis, MO, USA, 1∶1,000) and anti tyrosine hydroxylase rabbit antibody (AB152, Millipore, Billerica, MA, USA) Both, primary and secondary antibodies were complemented with 0.1% Triton X-100 and 1% BSA. No additional blocking step was performed. Incubation was done under a glass chamber (made of coverslips) in a humid box for 12–24 h at room temperature [27]. Washings between antibody incubations and after incubation with secondary antibodies were performed with PBS with 0.05% Triton X-100 at 37°C, 3×20 min. In order to stabilize preparations, immunostained sections were post-fixed with 2% paraformaldehyde for 10 min before counterstaining with DAPI (2 µg/ml) for 1 h and mounted in Vectashield medium (Vector Labs, Burlingame, CA, USA).

### Microscopy

Epifluorescence images were obtained on a Zeiss Axiovert 200 microscope equipped with Plan-Apochromat ×63/1.4 numerical aperture (NA) oil immersion objective lenses and a Sensicam (PCO) CCD camera. Confocal images were collected using an UltraVIEW VoX spinning disc system (Perkin Elmer) on a Nikon Ti microscope equipped with an oil immersion Plan-Apochromat ×40/1.3 NA objective lens (pixel size in XY = 186 nm, Z-step = 0.3 µm).

Scoring of tyrosine hydroxylase and MeCP2 positive cells was done by eye in z-stacks.

## Results and Discussion

### Generation of rat/mouse monoclonal antibodies against MeCP2

To generate new rat and mouse antibodies potentially detecting different domains of MeCP2, we generated a baculovirus expression plasmid coding for the full length rat MeCP2 with a double strep-tag and transfected/infected Sf9 insect cells with this construct. The recombinant protein was purified using strep-tactin sepharose leading to a single band in SDS-PAGE analysis (Figure 1). The protein was used to immunize Lou/C rats and CBL mice, leading to the generation of a panel of clonal hybridomas by fusion of lymphocytes from immunized animals with the myeloma cell line P3X63-Ag8.653. All antibodies generated by the hybridomas were initially screened in a solid-phase enzyme linked immunosorbent assay (ELISA, data not shown). Positive hybridoma supernatants from clones 4H7, 4G10 and 4E1 (rat monoclonal) as well as 4B4 and 8D11 (mouse monoclonal) were stably subcloned and used for further characterization. In parallel, we immunized rabbits with untagged human MECP2 protein to generate polyclonal antibodies and used the resulting antiserum directly.

### Sensitivity of the rat and mouse antibodies

To test the sensitivity of the antibodies we performed slot blot analysis with native rat MeCP2-GFP protein. The protein was applied in decreasing amounts ranging from 25 ng down to 0.78 ng. All monoclonal antibodies showed clear signals down to 1.56 ng of native protein and the rat monoclonal antibody 4E1 was still able to detect 0.78 ng of MeCP2 protein (Figure 2). The rabbit anti MeCP2 polyclonal antiserum was also able to detect down to 0.78 ng of native protein (Figure 2). The last column contained 500 ng BSA as negative control and none of the antibodies reacted with it.

### Epitope mapping

To determine the binding domain of the new monoclonal antibodies within the MeCP2 protein, we used different constructs of GFP/YFP tagged MeCP2 expressed in mammalian cells. The cell lysates were analyzed by SDS-PAGE, blotted on a nitrocellulose membrane and incubated with the different antibodies. All fusions were expressed as controlled by incubation of the membranes with anti GFP mouse monoclonal antibody. The results (Figure 3 and S1A) show that the rat monoclonal antibodies 4G10 and 4H7 reacted against the C-terminus of MeCP2 and 4E1 against the N-terminus. Both mouse monoclonal antibodies 4B4 and 8D11 showed specific binding to the C-terminus. Since none of the antibodies detected the MBD domain, which is highly conserved in all MBD proteins no cross-reaction with these proteins is expected. Additionally, the polyclonal rabbit antibody detected all fragments except the TRD.

### Specificity and cross species reactivity

**Figure 4:**
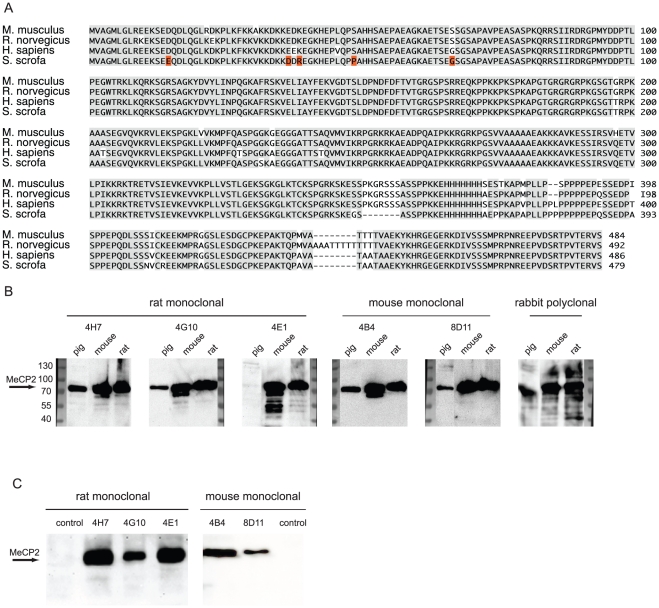
Antibody specificity. **A**) Sequence alignment of MeCP2 from different species. Identical residues are shaded in gray. The identities range from 93% (human-mouse) to 97% (rat-mouse). **B**) For a multi-species immunoblot nuclear extracts from pig, mouse and rat brain (10^6^ nuclei) were loaded and probed with the antibodies as indicated. **C**) For immunoprecipitation analysis, mouse brain whole cell (for rat antibodies) and nuclei (for mouse antibodies) extracts were incubated with the monoclonal antibodies as indicated followed by western blot analysis.

MeCP2 is highly conserved throughout different species (Figure 4A). To test for cross species reactivity, nuclei from pig, mouse and rat brain tissue were isolated and extracts analyzed by western blot. As shown in Figure 4B all antibodies detected endogenous MeCP2 in mouse and rat. Remarkably, the rat monoclonal antibody 4E1 was the only one that did not detect MeCP2 in nuclear extracts from pig brain. This coincides with the fact that it is the only antibody in our tests to react with the N-terminal part (amino acids 1–78) of MeCP2 (see below). Only five amino acids are not identical in this domain of MeCP2 in the pig compared to mouse and rat. We, thus, conclude that the epitope recognized by rat monoclonal antibody 4E1 must include one or more of these residues (Figure 4A, highlighted in red).

An important and commonly used method for studying protein interaction partners is immunoprecipitation. Thus, we tested next the ability of the monoclonal antibodies to specifically immunoprecipitate MeCP2 from mouse brains. We could show that our three rat antibodies were able to specifically pull down MeCP2 from whole brain extract (Figure 4C). Additional unspecific bands at 35 kDa were also detected in the negative control (rat anti RFP antibody mix) [26] and are most probably due to unspecific binding to the beads (data not shown). To successfully use our two mouse monoclonal antibodies for immunoprecipitation we needed to isolate nuclei from mouse brain first. We could then show that our mouse antibodies were also capable of specifically pulling down MeCP2 from mouse brain nuclei extracts (Figure 4C).

**Figure 5:**
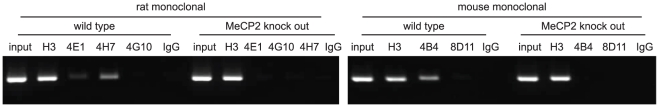
Chromatin immunoprecipitation. Chromatin immunoprecipitation assays were performed using mouse brain nuclear extracts obtained from wild type mice and MeCP2 knock out (KO) mice as negative control. The anti histone H3 antibody was used as a positive control of chromatin immunoprecipitation assay efficiency. IgG was used as a negative control of chromatin immunoprecipitation.

To next determine if our monoclonal MeCP2 antibodies are competent for chromatin immunoprecipitation analysis using purified nuclei from mouse brain. We analyzed the occupancy of MeCP2 in the promoter of *Xist* in the X chromosome, which is known to bind MeCP2 in mouse and is used as a standard positive control for MeCP2 binding. Two of our rat monoclonal antibodies were able to immunoprecipitate chromatin (4E1 and 4H7, Figure 5) with 4H7 producing a stronger signal than 4E1 and 4G10 not yielding a detectable signal. The latter might be due to technical limitations, or to the epitope recognized by the 4G10 mAb being masked when the MeCP2 protein is bound to chromatin. From our mouse antibodies only 4B4 was able to clearly chromatin immunoprecipitation (Figure 5). The three antibodies (rat 4E1 and 4H7 and mouse 4B4) suitable for chromatin immunoprecipitation show no band in the knockout brain whereas H3, which is used as positive control for chromatin immunoprecipitation, shows a band in wild type and knock out brain. Our polyclonal rabbit anti MeCP2 antibody was previously shown to be suitable for chromatin immunoprecipitation analysis [28]. Our antibodies therefore cover the whole range of important biochemical assays commonly performed.

### *In situ* analysis of MeCP2 in cells and in tissue

**Figure 6:**
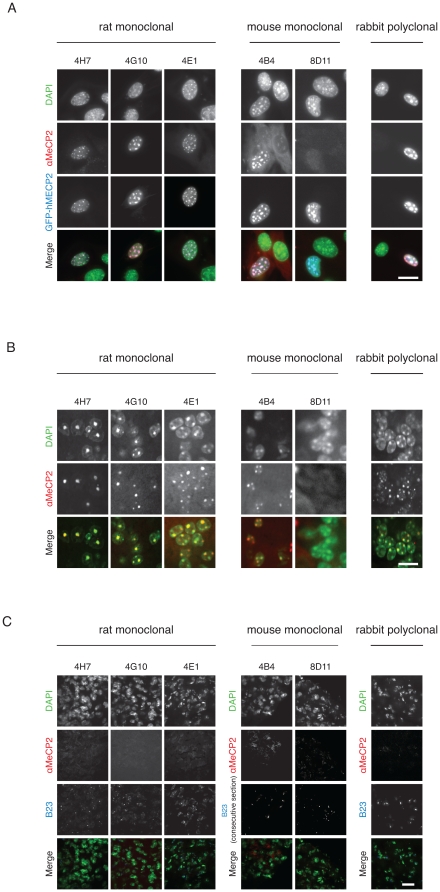
*In situ* analysis of MeCP2 in cells and tissue. **A**) Mouse myoblasts (C2C12 cells) were transiently transfected with GFP-MECP2 (human) and fixed using formaldehyde. MeCP2 was then detected with our monoclonal antibodies (undiluted) and our rabbit polyclonal antibody (1∶500). The first row shows the DNA counterstain (DAPI) of transfected and untransfected cells (green). The row underneath shows the signal obtained by our antibody staining (red). The third row shows the localization of the transfected GFP-MECP2 (blue). The merge contains an overlay of the antibody staining, the fluorescent signal of GFP-MECP2 and the DNA counterstain. Scale bar 20 µm. **B**) Mouse wild type brain sections (25 µm) were stained using our antibodies. The first row shows the DNA counterstain with DAPI highlighting heterochromatic regions. The central row shows the signal obtained by immunofluorescence with our antibodies. The last row shows an overlay of DAPI and MeCP2. Scale bar 20 µm. **C**) Mouse MeCP2 hemizygous null brain sections (25 µm) were stained as described above as a negative control. Mouse anti B23 antibody was used as a positive control (consecutive section when testing mouse monoclonal anti MeCP2). Scale bar 40 µm.

Western blot techniques usually deal with denatured protein and do not give information about the localization of the protein in the cell. It is therefore important to test whether the new antibodies correctly detect MeCP2 localization *in situ*. MeCP2 is predominantly localized at pericentric heterochromatic regions in mouse cells, which are highly enriched in strongly methylated major satellite DNA repeats and tend to form clusters known as chromocenters [14]. Immunostainings were thus performed on mouse myoblasts expressing GFP tagged human MECP2 using formaldehyde and methanol as fixation reagents (Figure 6A and S1B). Our three rat monoclonal antibodies revealed strong signals co-localizing with the ectopically expressed GFP-MECP2 and worked in both fixation conditions. Untransfected cells did not give a signal, consistent with undetectable endogenous levels of MeCP2 in those cells [14]. Using the mouse monoclonals, only 4B4 gave a signal for ectopically expressed protein. 8D11 exhibited high background noise and no specific binding in both fixation conditions. Our polyclonal rabbit antiserum showed strong and specific binding even when used at a dilution of 1∶500. DAPI was used as a counterstain and additional control in all cells since DAPI's preference for AT rich regions strongly highlights chromocenters.

Since MeCP2 plays a crucial role in RTT syndrome one of the most important goals for us was to test whether the antibodies work on brain tissue detecting MeCP2 in its native conformation. We, therefore, prepared cryosections of wild type mouse brain and also MeCP2 hemizygous null male mouse brain as negative control. The 25 µm-thick wild type brain sections were stained with the anti MeCP2 antibodies and counterstained with DAPI as marker for chromocenters. As demonstrated in Figure 6B our rat monoclonal antibodies show a strong and specific staining of chromocenters colocalizing with DAPI. Our mouse monoclonal antibody 4B4 shows a less intense but still specific staining of MeCP2. Unfortunately, the antibody 8D11 was not able to detect endogenous MeCP2 in brain, as it had failed to do with ectopic MeCP2 expression in cells, and is therefore not suitable for immunofluorescence. The strongest signals were achieved with our polyclonal rabbit antiserum, which was used as a positive control (Figure 6B). To verify the specificity to MeCP2 we performed the same stainings in MeCP2 null mouse brain sections. We added anti B23 nucleoli marker antibody as a positive staining control. As shown in Figure 6C none of the anti MeCP2 antibodies showed any significant signal in the knock out brain sections whereas B23 showed a clear and specific signal. The double staining with anti-B23 mouse monoclonal antibody was facilitated by the combination with our rat monoclonals, whereas, in the case of the mouse monoclonals, we had to perform the anti B23 staining using an adjacent tissue section. Additionally, using rat monoclonal antibodies obviated the cross reaction with endogenous mouse immunoglobulins present in the tissue, whereas these were readily detected when using the mouse monoclonal antibodies.

### X chromosome inactivation skewing in MeCP2 heterozygous mouse brain

Since MeCP2 lies on the X chromosome it is subjected to random X chromosome inactivation in early development. In RTT, X chromosome inactivation leads to a mosaic pattern of all the cells, theoretically in a 50∶50 ratio of healthy (active X containing wild type *MeCP2* allele) and affected (active X containing mutant *Mecp2* allele) cells. Deviations from this ratio indicate skewed inactivation of the X chromosome and affect the severity of RTT symptoms. Our antibodies should be highly suitable for studies concerning X chromosome inactivation as well as other studies on RTT affected brain and other tissues.

**Figure 7:**
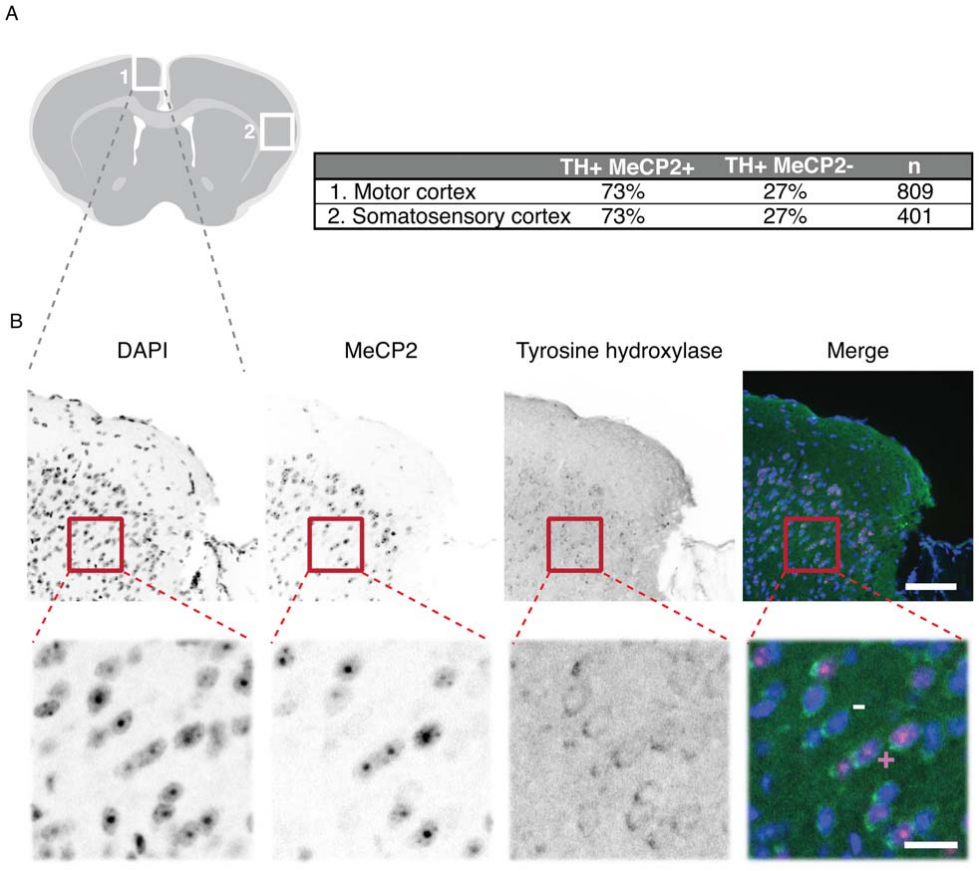
X chromosome inactivation skewing in brain from heterozygous MeCP2 null mouse. **A**) Schematic overview of a cryosection of a (female) heterozygous MeCP2 null brain with regions analyzed for X chromosome inactivation skewing marked with white squares. The results of the quantification of tyrosine hydroxylase positive, MeCP2 positive or negative cells are shown. N indicates the number of tyrosine hydroxylase positive neurons scored. **B**) Representative images of a section co stained with DAPI (DNA), anti MeCP2 (4H7) and anti tyrosine hydroxylase antibodies. The motor cortex region is depicted in an overview (upper panels; scale bar 80 µm). A magnification corresponding to the red square is shown in the lower panels (scale bar 20 µm). To illustrate the scoring strategy, an example of tyrosine hydroxylase and MeCP2 positive neuron is marked by+and of tyrosine hydroxylase positive and MeCP2 negative neuron is marked by -.

To test this, we evaluated X chromosome inactivation skewing in female MeCP2 heterozygous brain. We performed a double staining with rabbit anti tyrosine hydroxylase (TH) and our rat MeCP2 antibody (4H7). TH is the first enzyme in the biosynthesis of dopamine and norepinephrine from tyrosine and is, therefore, a marker for dopaminergic and noradrenergic neurons. Roux et al. [29] showed that TH positive cells always co-expressed MeCP2 and, hence, X chromosome inactivation skewing can be obtained by counting TH positive cells with and without MeCP2 signal. We focused on two areas of the cortex, the motor cortex and the somatosensory cortex (Figure 7). In both cases we could observe a pronounced X chromosome inactivation skewing favoring wild type MeCP2 expression (73%). Previously published mouse data suggest that X chromosome inactivation skewing in brain is the reason for very different phenotypes in RTT [30]. The degree of skewing is controversial and might dependent on the tissue analyzed or the method applied [30], [31]. Our antibodies could help to elucidate the state of X chromosome inactivation in RTT tissue in particular also with respect to truncated versus full length MeCP2 by a combination of the N and C-terminal specific antibodies.

**Figure 8:**
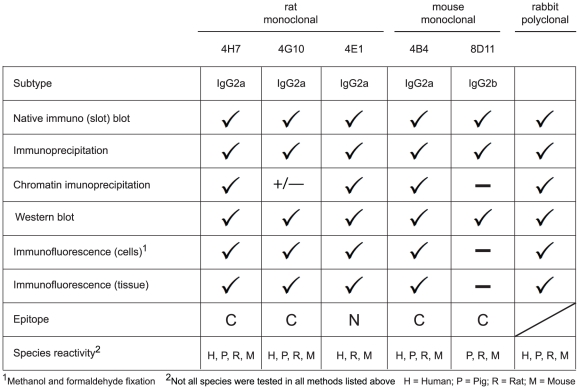
Summary of the characterization of the rabbit, rat and mouse anti MeCP2 antibodies.

Figure 8 summarizes the characterization of the novel anti MeCP2 antibodies. The antibodies recognize MeCP2 from different species, including human, mouse, rat and pig. Whereas the two new mouse antibodies are suitable for western blot, immunoprecipitation and to a lesser extend for immunofluorescence, the rabbit polyclonal as well as the rat monoclonal antibodies performed very well in immunoblotting, immunoprecipitation, and immunofluorescence analysis of ectopic and endogenous MeCP2. In addition, one mouse and two rat monoclonal antibodies as well as the rabbit polyclonal antiserum perform well in chromatin immunoprecipitation making them a very valuable set of tools for studies of MeCP2 pathophysiology *in situ* and *in vitro*.

## Supporting Information

Figure S1 **A) Epitope mapping.** Complete blots of the epitope mapping presented in Figure 3 together with a schematic representation of the constructs. **B)** ***In situ*** **analysis of MeCP2 in cells.** Mouse myoblasts (C2C12 cells) were transiently transfected with GFP-MECP2 (human) and fixed with methanol. MECP2 was then detected with our monoclonal antibodies (undiluted) and our rabbit polyclonal antibody (1∶500). The first row shows the DNA counterstain (DAPI) of transfected and untransfected cells (green). The row underneath shows the signal obtained by our antibody staining (red). The third row shows the localization of the transfected GFP-MECP2 (blue). The merge contains an overlay of the antibody staining, the fluorescent signal of GFP-MECP2 and the DNA counterstain. Scale bar 20 µm.
(PDF)
