# Supplementary material for: Generation and Characterization of Rat and Mouse Monoclonal Antibodies Specific for MeCP2 and Their Use in X-Inactivation Studies
Source: PLoS One. 2011 Nov 28;6(11):e26499. doi: 10.1371/journal.pone.0026499 (PMC3225355; doi:10.1371/journal.pone.0026499)
Supplement: Figure S1 — A) Epitope mapping. Complete blots of the epitope mapping presented in Figure 3 together with a schematic representation of the constructs. B) In situ analysis of MeCP2 in cells. Mouse myoblasts (C2C12 cells) were transiently transfected with GFP-MECP2 (human) and fixed with methanol. MECP2 was then detected with our monoclonal antibodies (undiluted) and our rabbit polyclonal antibody (1∶500). The first row shows the DNA counterstain (DAPI) of transfected and untransfected cells (green). The row underneath shows the signal obtained by our antibody staining (red). The third row shows the localization of the transfected GFP-MECP2 (blue). The merge contains an overlay of the antibody staining, the fluorescent signal of GFP-MECP2 and the DNA counterstain. Scale bar 20 µm. (PDF) [file pone.0026499.s001.pdf]

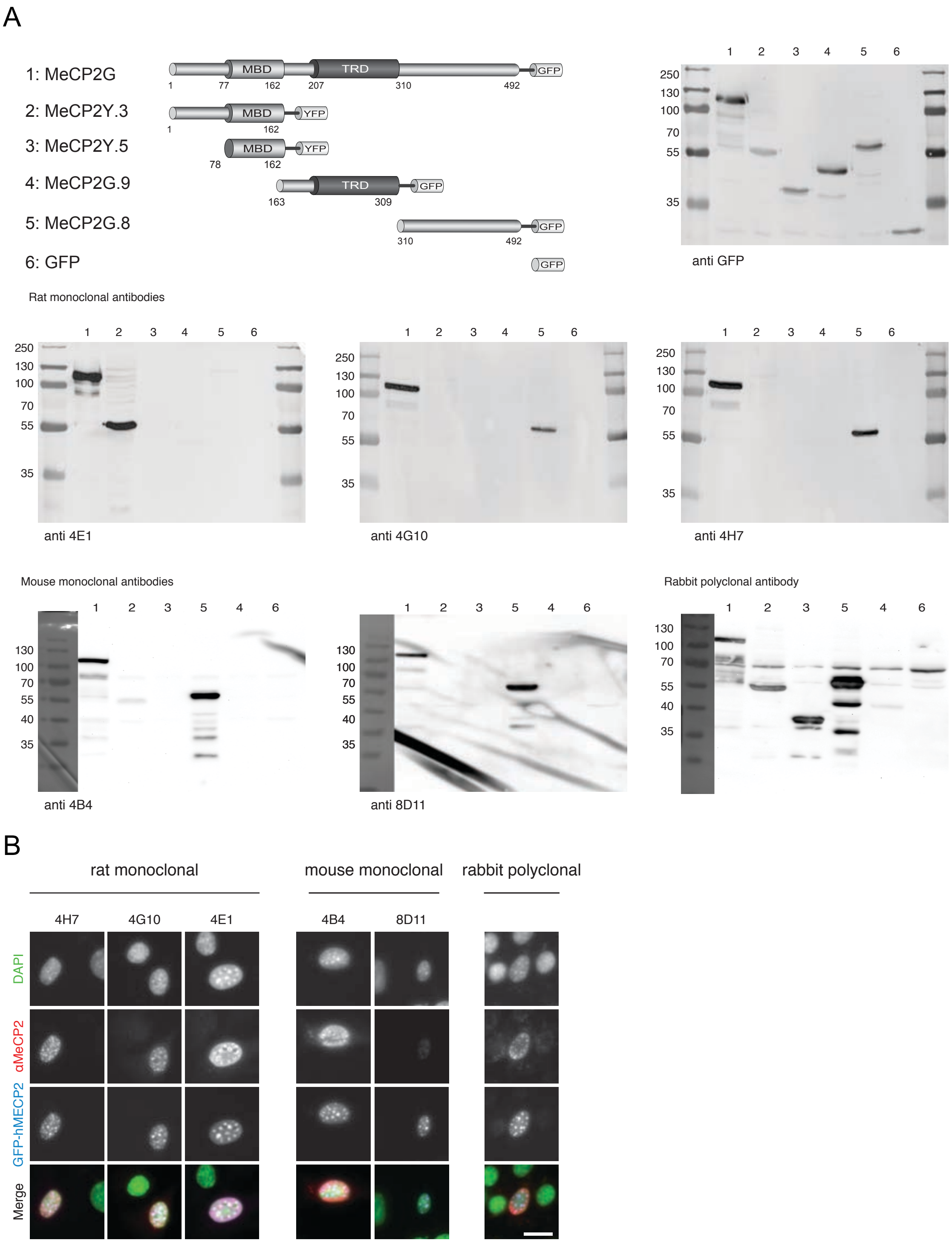

Figure S1. A) Epitope mapping. Complete blots of the epitope mapping presented in Figure 3 together with a schematic representation of the constructs. **B)** ***In situ*** **analysis of MeCP2 in cells.** Mouse myoblasts (C2C12 cells) were transiently transfected with GFP-MECP2 (human) and fixed with methanol. MECP2 was then detected with our monoclonal antibodies (undiluted) and our rabbit polyclonal antibody (1:500). The first row shows the DNA counterstain (DAPI) of transfected and untransfected cells (green). The row underneath shows the signal obtained by our antibody staining (red). The third row shows the localization of the transfected GFP-MECP2 (blue). The merge contains an overlay of the antibody staining, the fluorescent signal of GFP-MECP2 and the DNA counterstain. Scale bar 20 µm.
